# Hyperhemolysis Syndrome without Underlying Hematologic Disease

**DOI:** 10.1155/2015/180526

**Published:** 2015-02-17

**Authors:** Lauren Anne Eberly, Diaa Osman, Nathaniel Perryman Collins

**Affiliations:** Department of Internal Medicine, University of New Mexico Health Sciences Center, Albuquerque, NM 87131, USA

## Abstract

*Introduction*. Hyperhemolysis is characterized by a life-threatening hemolytic transfusion reaction, with hemoglobin (Hb) and hematocrit (Hct) dropping markedly lower than before transfusion. This phenomenon, commonly described in sickle cell disease, is a rare occurrence in patients without hemoglobinopathies. *Case Report*. A 55-year-old male presented to the hospital after a motorcycle crash and received 10 units of cross-matched blood for active bleeding. The patient was blood group O, with a negative antibody screen. Ten days later, he represented complaining of dyspnea and was found to have a hematocrit of 12%. The direct antiglobulin test was positive for anti-immunoglobin G and complement. Indirect antiglobulin test was positive for anti-Jka alloantibodies. The presence of Jka antigen was revealed in one unit of previously transfused blood; patient's RBCs were negative for the Jka antigen. Laboratory data demonstrated findings consistent with DHTR, as well as reticulopenia and elevated ferritin levels. He continued to show signs of active hemolysis, requiring a total of 4 subsequent units of pRBCs. Each transfusion precipitated a drop in Hb and Hct to levels lower than before transfusion; once transfusions were held, the patient slowly recovered. *Discussion*. Hyperhemolysis in the setting of a DHTR can occur in patients without hematologic disease.

## 1. Background

Hyperhemolysis is characterized by a hemolytic transfusion reaction that leads to a life-threatening anemia, with drops in hemoglobin (Hb) and hematocrit (Hct) to levels markedly lower than those present before transfusion. This phenomenon has been commonly described in sickle cell disease [1–7] and B-thalassemia major [8–10] but is an exceedingly rare occurrence in patients without hemoglobinopathies. Here we present the case of suggested hyperhemolysis in a patient without any underlying hematologic disorder.

## 2. Case Report

The patient was a 55-year-old male, who presented to the emergency department (ED) after sustaining multiple fractures of all four extremities in a motorcycle crash. He received a total of 10 units of packed red blood cells for active bleeding. Chart review revealed patient had normal levels of hemoglobin and hematocrit prior to his accident. He was discharged to a rehabilitation center, but ten days later, the patient presented again to the ED, complaining of severe dyspnea and fatigue. Physical exam revealed a systolic flow murmur with hyperdynamic precordium; otherwise, exam was unchanged from his previous discharge.

Laboratory evaluation showed Hb and Hct at 5.4 g/dL and 15%, respectively, and evidence of hemolysis with lactate dehydrogenase at 2355 U/L (normal range, 117–224 U/L), total bilirubin at 5.9 mg/dL (normal range, 0.3–1.2 mg/dL) with indirect bilirubin at 4.3 mg/dL (normal range, 0.2–1.0 mg/dL), and haptoglobin < 8 mg/dL (normal range, 30–200 mg/dL). Plasma hemoglobin was elevated at 11.1 mg/dL (normal range, 0.5–5 mg/dL). Patient passed dark-colored urine, and urine analysis confirmed the presence of hemoglobin. Further work-up revealed a positive direct antiglobulin test (DAT) with 3+ reactivity for both IgG and complement. Indirect antiglobulin test (IAT) was positive, demonstrating the presence of anti-Jka alloantibodies. Patient's RBCs were phenotyped and found to be Jka negative. Further history obtained at this time revealed that the patient had received a blood transfusion three decades before.

On day one, the patient was transfused with 2 units of Jka negative pRBCs. His hemoglobin and hematocrit initially rose to 6.1 g/dL and 16% directly after the transfusion but within 5 hours were lower than those before transfusion, with a value of 5.0 g/dL and 14%. On day two, the patient's hemoglobin had dropped further to 4.6 g/dL (Hct 13%), and he was transfused again with 1 unit of Jka negative blood. Again, his Hb and Hct rose directly after transfusion to 5.8 g/dL and 17% but then continued to fall. In four hours, Hb dropped to 5.4 g/dL (Hct 15%), and thus another unit of Jka negative pRBCs was transfused. Subsequent Hb and Hct were 5.3 g/dL and 15%, respectively, after the transfusion. On the morning of day four, repeat Hb and Hct were 4.3 g/dL and 12%. All Jka negative blood units transfused were compatible after cross-matching with patient's serum. A new blood sample on day 3 showed persistent DAT positivity, with continued 3+ reactivity to IgG and complement. IAT remained positive due to anti-Jka alloantibody, but no additional alloantibodies or autoantibodies were identified on repeat testing.

Poor reticulocyte response was found with reticulocyte count to be at 5.3% (normal range, 0.5%–2.5%) and reticulocyte index at 0.7. Ferritin was elevated at 6298 *μ*g/L (normal range, 8–252 mg/dL). B12 and folate levels were normal, as were coagulation studies. Peripheral smear showed nucleated RBCs and spherocytes. Subsequent evaluation indicated absence of cold agglutinins, normal glucose-6-phosphate dehydrogenase and pyruvate kinase activity, and absence of any underlying hemoglobinopathy. Patient was guaiac negative, with no significant nasogastric tube findings. CT of the abdomen and pelvis was also performed, which was negative for any active bleeding. Blood cultures were finalized as negative.

Further transfusions were held due to the continued dropping Hb and Hct with each subsequent transfusion. Hematology was consulted and deferred a bone marrow examination. Iron supplementation was given, and on day 16, his Hb and Hct had slowly increased to a steady level of 8.2 g/dL and 24%. Patient was subsequently discharged to a rehabilitation facility. Reticulocyte count was improved on day of discharge at 10.6%; ferritin level was not repeated prior to discharge.

## 3. Methods and Results

Automated reticulocyte counting (Automated Hematology Analyzers, Flurocell RET, Sysmex, Mundelein, IL) was used. ABO blood grouping and Rh(D) phenotyping were performed with reagents from Immucor (Norcross, GA). The antibody screen was performed with three reagent RBCs from Immucor (Raritan, NJ). Antibody identification was performed with identification panels from Immucor and ONE Gamma Biologicals, Inc. (Houston, TX). The DAT was performed with reagents from Immucor. Samples reactive with a polyspecific reagent were repeated with monospecific anti-IgG and a blend of anti-C3b and anti-C3d. Acid eluates were prepared with an elution kit from Immucor. The final wash, run in conjunction with the eluate, showed reactivity to IgG and C3. The patient's RBCs were phenotyped with reagents from Ortho-Clinical Diagnostics, Inc. (Raritan, NJ) and Biorad Medical Diagnostics (Dreieich, Germany) for D, C, c, E, e, K, k, Fya, Fyb, Jka, Jkb, S, and s. The previously transfused donor RBC unit of interest was phenotyped with the same method.

## 4. Discussion

Hyperhemolysis is characterized by destruction of both transfused and autologous RBCs, which leads to a consequent fall in posttransfusion hemoglobin lower than the level present before transfusion [3, 11, 12]. Both an acute and delayed form of hyperhemolysis has been established, and while acute hemolytic transfusion reactions and acute hyperhemolysis can be easily differentiated, the distinction between the delayed entities is less clear.

In delayed hemolytic transfusion reactions (DHTR), alloantibody formation is present, leading to destruction of transfused RBC only. Delayed hyperhemolysis, a suggested subset of DHTR, involves the delayed destruction of both transfused and autologous cells [4]. Our patient, although initially thought to have developed a DHTR, had posttransfusion Hb and Hct levels that were consistently lower than those present before transfusion, which is more consistent with hyperhemolysis. Additionally, transfusions in a hyperhemolytic episode can accelerate hemolysis causing life-threatening anemia, which was seen in our case. Jka negative blood products potentiated drops in the Hb and Hct in our patient, suggesting that autologous RBC destruction was occurring.

In hemolytic reactions, reticulocytosis is normally marked, as the bone marrow attempts to compensate for dropping blood levels. However, while formed alloantibodies may or may not be seen in delayed hyperhemolysis [6], reticulopenia has been well documented as a manifestation of hyperhemolysis [6, 12]. Although previously believed to be secondary to suppression of erythropoiesis from multiple transfusions, it is now hypothesized that this is likely due to peripheral consumption and destruction of reticulocytes by macrophages [3, 6]. It has been suggested that antibodies reacting with foreign antigens on transfused cells can cause activation of complement, leading to a phenomenon known as “bystander hemolysis” [13]. This is supported by the fact that IVIG and steroids, which can block immune destruction, increase blood reticulocytes in patients undergoing a hyperhemolytic reaction [6, 14]. The observed reticulopenia in our patient, contrary to what was expected for DHTR, suggests that consumption of reticulocytes was occurring. Hyperhemolysis is further suggested by the improvement of the reticulocyte count at discharge, along with the high ferritin level present during hemolysis.

The pathophysiology of hyperhemolytic syndrome is not well established. This case further reveals its complexity as it suggests that the mechanism of autologous destruction is irrespective of defective hemoglobin or hematologic abnormality. There are few clinical reports of hyperhemolysis in patients without hemoglobinopathy; this syndrome has been demonstrated in patients with myelofibrosis [15] and anemia of chronic disease [16] and dyserythropoietic anemia [17] (CDA) type I and also rarely in patients with underlying hematologic malignancies such as chronic lymphocytic leukemia [18], mantle cell lymphoma [19], and marginal cell lymphoma [20]. Our case demonstrates the possibility of hyperhemolysis in the setting of a DHTR in patients without hematologic disease. Anti-Jka causes over one-third of DHTRs, which are often severe and fatal [21]. Whether Jk antibodies are more likely to precipitate hyperhemolysis remains to be determined.

This case emphasizes the importance of clinicians to be aware of the potential of hyperhemolysis in patients without hematologic disease, who develop transfusion reactions. Quicker recognition can allow for more abrupt cessation of exacerbating transfusions and for steroid and IVIG administration and thus quicker resolution. Our patient did not receive IVIG or steroid treatment due to our unfamiliarity with the possibility of hyperhemolysis in patients without SCD or thalassemia. We suspect that the administration of these therapies would have led to prompter resolution of the patient.

## 5. Conclusions

Hyperhemolysis syndrome in the setting of a DHTR can occur in patients without underlying hematologic disease; the presence of this syndrome should be recognized promptly to allow for appropriate management.
